# OTS167 blocks FLT3 translation and synergizes with FLT3 inhibitors in *FLT3* mutant acute myeloid leukemia

**DOI:** 10.1038/s41408-021-00433-3

**Published:** 2021-03-03

**Authors:** Bartholomew J. Eisfelder, Caner Saygin, Joseph Wynne, Margaret W. Colton, Mariafausta Fischietti, Elspeth M. Beauchamp, Jason X. Cheng, Olatoyosi Odenike, Gail Roboz, Houda Alachkar, Wendy Stock

**Affiliations:** 1 Section of Hematology/Oncology, Department of Medicine, University of Chicago, Chicago, IL USA; 2 Department of Hematology/Oncology, Robert H Lurie Comprehensive Cancer Center, Northwestern University, Chicago, IL USA; 3 Department of Pathology, University of Chicago, Chicago, IL USA; 4 Weill Cornell Medicine, The New York Presbyterian Hospital, New York, NY USA; 5 Department of Clinical Pharmacy, School of Pharmacy, University of Southern California, Los Angeles, CA USA

**Keywords:** Acute myeloid leukaemia, Cell signalling

## Abstract

Internal tandem duplication (-ITD) mutations of Fms-like tyrosine kinase 3 *(FLT3)* provide growth and pro-survival signals in the context of established driver mutations in *FLT3* mutant acute myeloid leukemia (AML). Maternal embryonic leucine zipper kinase *(MELK)* is an aberrantly expressed gene identified as a target in AML. The MELK inhibitor OTS167 induces cell death in AML including cells with *FLT3* mutations, yet the role of MELK and mechanisms of OTS167 function are not understood. OTS167 alone or in combination with tyrosine kinase inhibitors (TKIs) were used to investigate the effect of OTS167 on FLT3 signaling and expression in human *FLT3* mutant AML cell lines and primary cells. We describe a mechanism whereby OTS167 blocks FLT3 expression by blocking FLT3 translation and inhibiting phosphorylation of eukaryotic initiation factor 4E–binding protein 1 (4E-BP1) and eukaryotic translation initiation factor 4B (eIF4B). OTS167 in combination with TKIs results in synergistic induction of *FLT3* mutant cell death in *FLT3* mutant cell lines and prolonged survival in a *FLT3* mutant AML xenograft mouse model. Our findings suggest signaling through MELK is necessary for the translation and expression of FLT3-ITD, and blocking MELK with OTS167 represents a viable therapeutic strategy for patients with *FLT3* mutant AML.

## Introduction

Approximately 30% of cases of acute myeloid leukemia (AML) aberrantly express a mutated Fms-like tyrosine kinase 3 (*FLT3*) gene characterized by an internal tandem duplication (ITD), point mutations within the juxtamembrane (JM) domain, or point mutations in the tyrosine kinase domain (TKD)^1^. Constitutive signaling mediated by mutant *FLT3* results in anti-apoptotic and pro-growth signals that are critical for the progression of *FLT3* mutant AML^2–6^. Patients with *FLT3* mutant AML typically exhibit worse clinical outcomes although recent phase III data demonstrate a survival improvement when a targeted kinase inhibitor (midostaurin) is added to combination chemotherapy, followed by allogeneic transplant in first complete remission^7–9^. Targeting the FLT3 kinase, midostaurin has been approved for the treatment of newly diagnosed *FLT3* mutant AML; gilteritinib has been approved recently for relapsed/refractory (R/R) AML^10,11^. Results from the ADMIRAL Phase III study (NCT02421939) show significantly longer overall survival (OS) and higher response rates with gilteritinib vs salvage chemotherapy in patients with R/R AML. Yet, the median OS for patients in the gilteritinib arm is less than one year, thus additional therapeutic options are required^12^. Despite the availability of these tyrosine kinase inhibitors (TKIs) for the treatment of *FLT3* mutant AML, clonal evolution or drug resistance may result in failure of TKI activity. The acquisition of additional FLT3 mutations during treatment represents a mechanism by which patients acquire resistance to TKIs^13,14^. Therefore, combination therapies offer a strategy to contend with multiple mutations and other mechanisms of TKI resistance in mutant *FLT3* leukemia.

Maternal embryonic leucine zipper kinase (MELK) is a serine/threonine protein kinase that is aberrantly expressed in many tumor types and demonstrated to be important for the formation and maintenance of cancer stem cells^15,16^. We have previously reported that MELK is aberrantly expressed in AML cell lines and primary patient leukemia cells, and is associated with a poor prognosis^17^. MELK knockdown, or inhibition using small-molecule MELK inhibitor OTS167, blocked the growth of several AML cell lines including cells with *FLT3* mutations. Nanomolar doses of OTS167 induced cell death in primary patient samples aberrantly expressing mutant FLT3^17^. Currently, we are testing OTS167 in a Phase I Clinical Trial (NCT02795520) for patients with relapsed/refractory AML^18^. Here, we investigate the antileukemia activity of OTS167 in *FLT3* mutant AML alone or in combination with TKIs. We describe a mechanism whereby MELK inhibition targets *FLT3* mutant AML through inhibition of FLT3-ITD signaling and downregulation of FLT3-ITD expression. We further demonstrate the synergy of OTS167 with FLT3 kinase inhibitors in *FLT3* mutant AML cell lines and in a xenograft mouse model of *FLT3* mutant AML.

## Materials and methods

### Viability assays, CFC assays, and synergy analysis

Cell viability assays were performed on cells incubated for 48 h in the presence of increasing concentrations of single or combination drug treatments. Cell viability was assayed using Cell Counting Kit-8 (CCK-8)(Dojindo Molecular Technologies, Inc, Rockville, MD) added to the cell culture for the last 4 h, and quantitated using a Bio Tek Synergy H4 plate reader using Gen5 software (SCR_017317). The half-maximal inhibitory concentration (IC50) for cell viability assays was calculated using nonlinear best fit [Inhibitor] vs. response—variable slope (four-parameter) in GraphPad Prism v.8.0 (RRID: SCR_002798). Colony forming cell (CFC) assay was performed by plating 5E4 primary blasts in 0.9% MethoCult (#H4434, StemCell Technologies, Vancouver, Canada). Cultures were incubated at 37degC in a humidified atmosphere of 5% CO_2_ for 10–14 days. All types of colonies were counted and labeled as #CFU. Experiments were done in duplicate or triplicate. Synergy analysis using Combination Index (CI) for different dose combination cell viability percentages (Fa) was calculated using CompuSyn^19,20^. A combination index value below 1 represents synergistic induction of cell death.

### Animal xenograft studies

Animal studies were approved by the University of Chicago Institutional Animal Care and Use Committee and carried out with adherence to all appropriate guidelines and using a complication scoring system to minimize animal suffering. Xenograft experiments were performed as previously described^21^. Briefly, for MV4:11 xenograft experiments, NOD.Cg *Prkdc*^*scid*^*IL2rg*^*tm1Wjl*^/SzJ (NSG) mice (Jackson Laboratories, Bar Harbor, Maine) were serially engrafted via tail vein injection with 10E6 MV4:11, then 5E6 splenocytes from the initial engraftment (first generation), before engrafting 1E6 cells (second generation) for pharmacodynamic and survival experiments. 10 days after engraftment, engrafted animals were treated for the indicated times on a 5 days treated/2 days off schedule. For MOLM-14 xenograft experiments, NSG mice were engrafted directly with 1E6 MOLM-14 cells for pharmacodynamic and survival experiments. 7 days after engraftment, engrafted animals were treated for the indicated times on a 5 days treated/2 days off schedule. Inhibitors in powder form were suspended in 0.5% methylcellulose (Sigma #M0512) fresh each day for delivery via oral gavage.

### Statistical analysis

Statistical analysis was conducted using GraphPad Prism, v8.0. Grouped data are presented as mean ± SD. Differences between the two groups were assessed using a two-tailed unpaired *t* test. Survival outcomes were compared using the Kaplan–Meier method and the log-rank test. Replicates per group for survival analysis were based upon sample size calculations (predicted increase in life span of 4 days with combination therapy (~46 day life span with single drug alone); alpha = .05, beta=0.8, hazard ratio of 0.237)—10 animals per group were proposed. *P* values <0.05 are considered statistically significant. *P* value scheme: ****P < 0.0001, ***P < 0.001, **P < 0.01, *P < 0.05, ns-not significant.

Additional methods and figures can be found in Supplemental Materials.

## Results

### OTS167 downregulates FLT3 protein expression in *FLT3* mutant AML

**Fig. 1:**
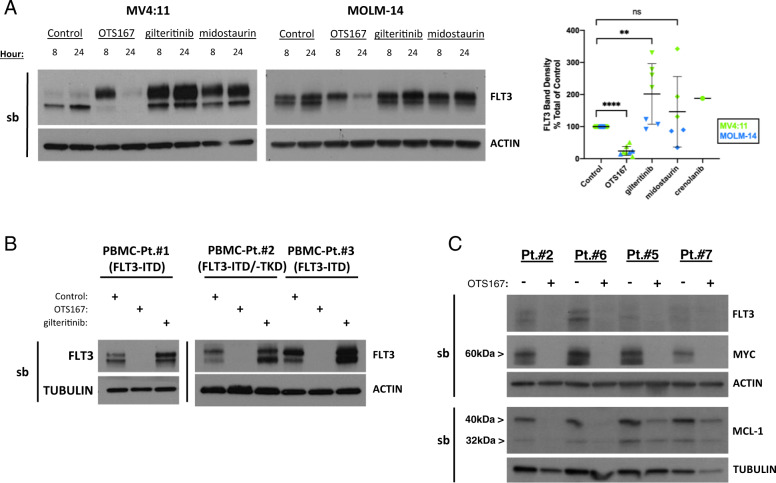
OTS167 downregulates FLT3-ITD expression in *FLT3* mutant cell lines and primary patient cells. **A** Western blot analysis of FLT3 expression in MV4:11 or MOLM-14 after treatment with 50 nM OTS167, 50 nM gilteritinib, or 50 nM midostaurin for 8 or 24 h. (single blot (sb)) Graph of seven biological replicates shows densities of both FLT3 bands together at 24 h as a percent total of control. (In graph, 50 nM crenolanib was also used to treat MV4:11). **B** Western blot analysis of FLT3 expression in *FLT3* mutant primary patient mononuclear cells after treatment with 50 nM OTS167 or 50 nM gilteritinib for 24 h. (1 biological replicate per patient)(single blot). **C** Western blot analysis of oncoprotein expression in *FLT3* mutant primary patient mononuclear cells after treatment with 50 nM OTS167 for 24 h. (1 biological replicate per patient)(single blots as indicated) (****P < 0.0001, **P < 0.01, ns-not significant).

OTS167 effectively induces cell death in human AML cell lines expressing FLT3-ITD and FLT3-TKD mutations^17^. To determine whether MELK plays a role in regulating FLT3 expression, FLT3-ITD-expressing cell lines MV4:11 or MOLM-14 were treated with OTS167 and assayed for FLT3 protein expression at 8 and 24 h. Western blots of untreated cells expressing FLT3-ITD protein reveal two protein bands corresponding to a simply glycosylated 130 kDa form (p130) retained in the endoplasmic reticulum (ER) and a complexly glycosylated 160 kDa form (p160) found on the cell surface, each regulating distinct signal transduction pathways^22^. Treatment of MV4:11 or MOLM-14 cells with 50 nM OTS167 resulted in a loss of the p130 form of FLT3 at 8 h and loss of both forms by 24 h (Fig. 1A – western blots). Quantitation of combined band densities (p130 and p160) showed a significant (****P < 0.0001) decrease in FLT3-ITD detection at 24 h after OTS167 treatment (Fig. 1A – graph). Expression of wild type FLT3 in human AML THP-1 cells was also downregulated by OTS167 (Fig. S1). Notably, treatment with FLT3 inhibitors gilteritinib or midostaurin resulted in increased FLT3 protein particularly the p160, consistent with previous studies^23^. Treatment of primary human *FLT3* mutant AML blast mononuclear cells (FLT3-ITD) with 50 nM OTS167 also resulted in loss of FLT3 protein expression at 24 h (Fig. 1B, C and Table 1). To determine whether OTS167 downregulated the expression of other oncoproteins implicated in AML, expression of c-Myc and MCL-1 was also assessed after 50 nM OTS167 treatment in primary human *FLT3* mutant cells (Fig. 1C and Table 1). Interestingly, c-Myc and MCL-1 expression were both downregulated by OTS167, suggesting a common mechanism that may block expression of aberrantly expressed oncoproteins.

**Table 1:** Patient information for primary cell samples.

*PB* peripheral blood, *BM* bone marrow, *ITD* internal tandemduplication, *TKD* tyrosine kinase domain, *HSCT* hematopoietic stem cell transplantation, *normal k.* normal karyotype.

### Translation of FLT3 protein is inhibited by OTS167 in FLT3-ITD-expressing cell lines

**Fig. 2:**
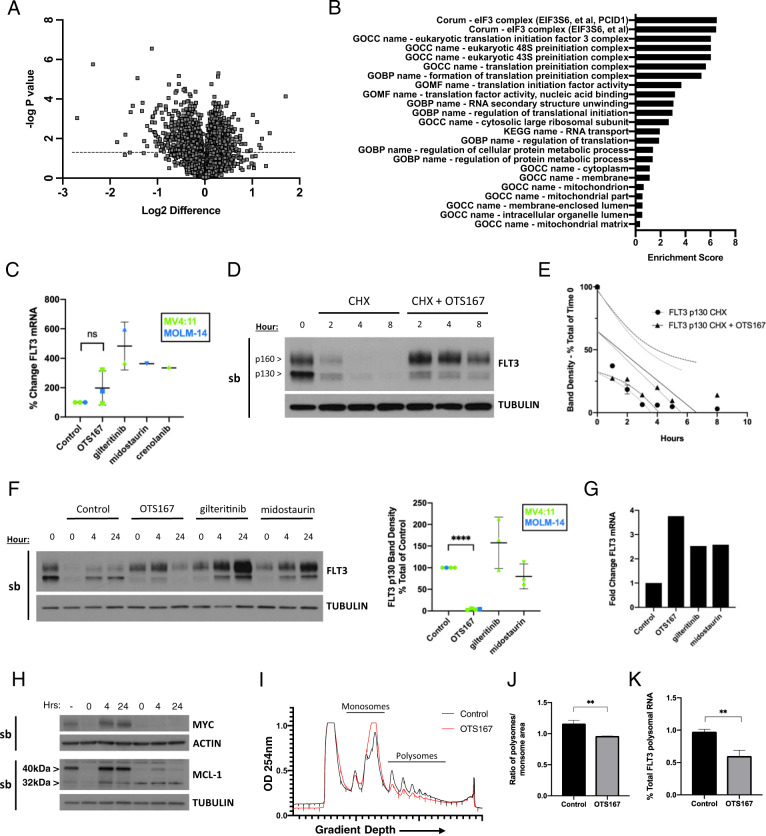
Downregulation of FLT3 protein expression due to inhibition of FLT3 translation. **A** Volcano plot of quantified proteins for MV4:11 treated with 50 nM OTS167 for 8 h, graphed by log2 change (x-axis) and -log P value (y-axis). Threshold for determining significant expression changes is indicated by a dashed line (*P* < 0.05). **B** Enrichment analysis of downregulated proteins in MV4:11 treated with 50 nM OTS167 for 8 h. Displayed is an aggregation of Gene Ontology (Cellular Component (GOCC), Biological Process (GOBP), Molecular Function (GOMF)), Corum, and Kyoto Encyclopedia of Genes and Genomes (KEGG) enrichment scores. Enrichment scores were calculated using -log P value. **C** Fold change in *FLT3* mRNA assayed by qPCR in MV4:11 or MOLM-14 after treatment with 50 nM OTS167, 50 nM gilteritinib, 50 nM midostaurin, or 50 nM crenolanib for 24 h. (3 biological replicates; 3 technical replicates each). **D** Western Blot analysis of FLT3 expression in MV4:11 over time after treatment with 100 ug/mL cycloheximide or 100 ug/mL cycloheximide plus 50 nM OTS167. (2 biological replicates)(single blot). **E** Band quantitation of Fig. 2D. Band values for each time point are normalized first to tubulin in each lane, then to 0 h time point. The difference between the linear regression curves (shown with dashed 95% confidence interval) were not statistically significant. **F** Western blot analysis of nascent FLT3 protein expression in MV4:11 at 4 and 24 h. MV4:11 were treated with 100 ug/mL cycloheximide and indicated drug (50 nM OTS167, 50 nM gilteritinib or 100 nM midostaurin) for 3 h, washed, then treated with same indicated drug (50 nM OTS167, 50 nM gilteritinib, or 100 nM midostaurin) for 4 or 24 h. (3 biological replicates)(single blot) Graph of four biological replicates (MV4:11 or MOLM-14) shows density of p130 FLT3 band as a percent total of control. **G** Fold change in *FLT3* mRNA assayed by qPCR in MV4:11 at 24 h. (1 biological replicate; 3 technical replicates). **H** Western blot analysis of nascent c-Myc and MCL-1 protein expression in MV4:11 at 4 and 24 h as described in Fig. 2F. (1 biological replicate)(single blots as indicated). **I** Representative OD254 spectra of cell lysates fractionated to separate monosomes and polysomes in control MV4:11 cells or MV4:11 cells treated with 50 nM OTS167 for 4 h. (2 control biological replicates, 3 OTS167 biological replicates). **J** Ratio of quantitative measurements (area under the curve) of pooled monosomal and pooled polysomal peaks using ImageJ. (2 control biological replicates, 3 OTS167 biological replicates). **K** Fold change of *FLT3* mRNA in pooled polysomal fractions assayed by qPCR. (3 biological replicates each) (****P < 0.0001, **P < 0.01, ns-not significant).

To further characterize the effect of OTS167 on protein expression in FLT3-ITD-expressing cells, a non-targeted proteomic analysis was performed to quantitate the levels of abundant proteins expressed in MV4:11 cells treated with 50 nM OTS167 for only 8 h. A volcano plot of quantified proteins shows the change in expression of proteins from OTS167-treated cells compared to control cells (Fig. 2A). Enrichment analysis of the significantly downregulated proteins at 8 h showed a substantial effect of OTS167 on protein translation, most dramatically diminishing the expression of proteins comprising the translation initiation factor 3 (eIF3) complex (Fig. 2B). The proteomic data suggested inhibition of translation caused the decrease in FLT3 expression. Nevertheless, to exclude other potential mechanisms that may affect *FLT3* transcription or mRNA stability, MV4:11 or MOLM-14 cells were treated with 50 nM OTS167 for 24 h, then assayed for *FLT3* mRNA (Fig. 2C). OTS167 treatment did not decrease *FLT3* mRNA levels compared with control cells, suggesting that OTS167 does not downregulate FLT3-ITD expression through inhibition of *FLT3* transcription or de-stabilization of *FLT3* mRNA. Furthermore, the loss of FLT3-ITD protein was not due to increased FLT3-ITD degradation; MV4:11 or MOLM-14 were treated with cycloheximide alone or in combination with OTS167 (Fig. 2D). Cells treated with cycloheximide and OTS167 did not lose the 130 kDa form of FLT3-ITD protein expression more rapidly than cells treated with cycloheximide alone, suggesting that OTS167 does not increase degradation of FLT3-ITD (Fig. 2E). To test proteosome-mediated degradation directly, we assessed whether the proteosome inhibitor bortezomib could block the downregulation of FLT3-ITD by OTS167 in MV4:11 or MOLM-14 cells (Fig. S2). Bortezomib failed to block the downregulation of FLT3-ITD by OTS167, further evidence that argues against increased degradation as a mechanism for FLT3-ITD downregulation. We next confirmed that OTS167 specifically blocked translation of the FLT3-ITD protein. First, new protein translation in MV4:11 cells was reversibly blocked with cycloheximide, resulting in depletion of the cellular FLT3-ITD protein pool. After washout of cycloheximide, new protein translation of FLT3-ITD (p130 ER form) could be observed (Fig. 2F - Control). Conversely, after depletion of cellular FLT3-ITD protein and subsequent treatment with 50 nM OTS167, newly translated FLT3-ITD protein (p130) was not detected after 24 h (Fig. 2F) despite the presence of *FLT3* mRNA (Fig. 2G). However, nascent FLT3-ITD protein (p130) expression was observed in cells treated with gilteritinib or midostaurin. Quantitation of p130 band densities shows a significant (****P < 0.0001) loss of nascent FLT3-ITD protein expression (Fig. 2F – graph). The p160 form of FLT3-ITD is not entirely depleted during cycloheximide treatment due to its stabilization by TKI treatment^24^. Supporting these results, nascent expression of cap-dependent translation oncoproteins c-Myc and MCL-1 was also blocked by 50 nM OTS167 (Fig. 2H). To further establish the inhibition of *FLT3* mRNA translation, MV4:11 cells treated with 50 nM OTS167 for 4 h were fractionated to quantitate *FLT3* mRNA-containing polysomes. Treatment of MV4:11 cells with OTS167 for even this short amount of time resulted in an increase in monosomal fractions and a decrease in polysomal fractions (Fig. 2I) similar to profiles observed with inhibitors targeting CDK9 or PIM kinase^25,26^. The ratio of polysomes/monosomes was significantly (**P < 0.01) decreased by treatment with OTS167, reflecting an inhibition of translation versus a loss of mRNA for which the ratio would not change (Fig. 2J). Consistent with inhibition of FLT3 protein expression assayed by western blot, polysomal fractions of OTS167-treated cells contained a significantly (**P < 0.01) decreased amount of *FLT3* mRNA at 4 h, confirming a rapid inhibition of *FLT3*-specific mRNA translation (Fig. 2K).

### OTS167 inhibits phosphorylation of 4E-BP1 and eIF4B in *FLT3* mutant AML cell lines

**Fig. 3:**
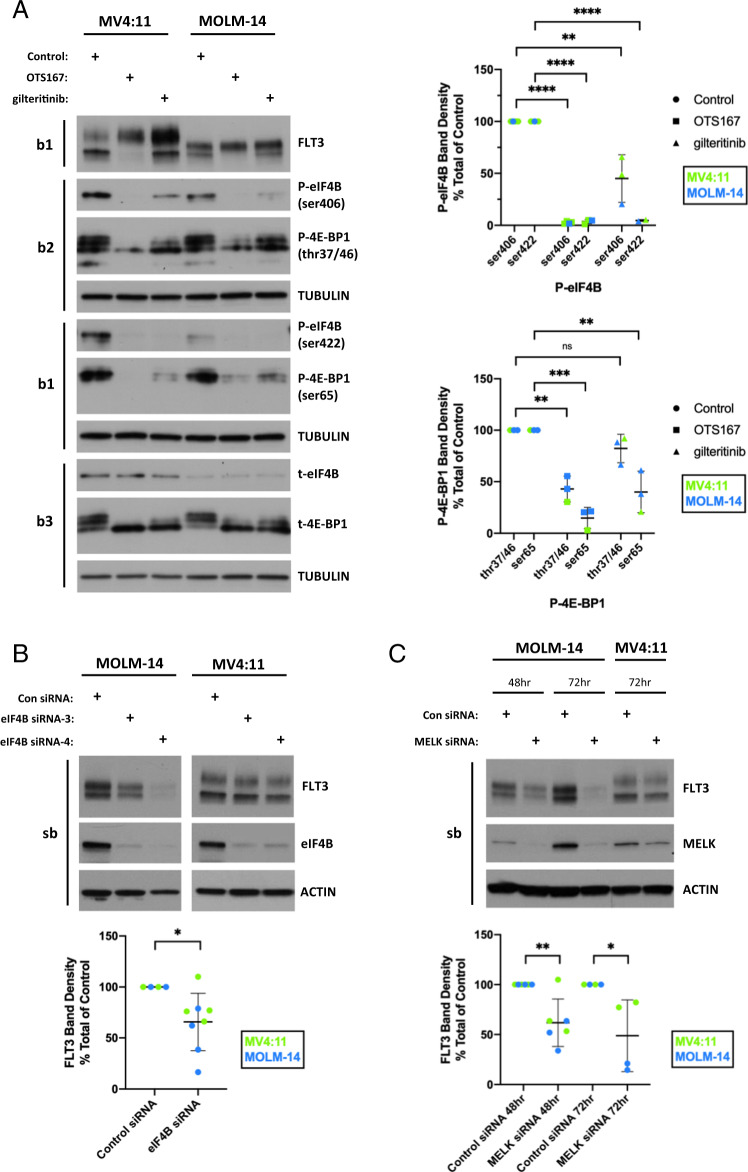
OTS167 inhibits eIF4B phosphorylation necessary for efficient FLT3 protein expression. **A** Western Blot analysis of P-eIF4B(ser406), P-eIF4B(ser422) or t-eIF4B (eIF4B species detected in parallel blots) or P-4E-BP1(thr37/46), P-4E-BP1(ser65) or t-4E-BP1 (4E-BP1 species detected in parallel blots) in MV4:11 or MOLM-14 after treatment with 50 nM OTS167 or 50 nM gilteritinib for 8 h. Graphs show densities of P-eIF4B (cells stimulated for 8 or 17 h) or P-4E-BP1 (cells stimulated for 8 h) bands as a percent of total protein (eIF4B or 4E-BP1) (5 biological replicates for P-eIF4B, 2 biological replicate for P-4E-BP1)(samples run in parallel on 3 blots(b1-3)) **B** Western Blot analysis of FLT3 and eIF4B expression in MOLM-14 or MV4:11 72 h after transfection with two separate eIF4B-siRNAs compared to Control-siRNA. (3 biological replicates)(single blot) Graph shows densities of both FLT3 bands as a percent total of control cells for MV4:11 or MOLM-14 72 h after transfection with either eIF4B-siRNAs. **C** Western Blot analysis of FLT3 and MELK expression in MOLM-14 or MV4:11 at 48 or 72 h after transfection with MELK-siRNA compared to Control-siRNA. (4 biological replicates)(single blot) Graph shows densities of both FLT3 bands as a percent total of control cells for MV4:11 or MOLM-14 transfected with MELK-siRNAs at 48 or 72 h. (****P < 0.0001, ***P < 0.001, **P < 0.01, *P < 0.05, ns-not significant).

Eukaryotic initiation factor 4E–binding protein 1 (4E-BP1) binds eukaryotic translation initiation factor 4E (eIF4E) based on the phosphorylation status of several 4E-BP1 sites including thr37/46 and ser65, and regulates the availability of eIF4E for cap-dependent mRNA translation^27–29^. Eukaryotic translation initiation factor 4B (eIF4B) facilitates translation of certain mRNA transcripts, and has previously been identified as a target for MELK activity^30,31^. eIF4B-mediated translation activity is positively regulated by phosphorylation of its ser406 and ser422 residues^30,32–34^. To determine if the block in translation of FLT3-ITD may be due to inhibition of 4E-BP1 or eIF4B, MV4:11 or MOLM-14 were treated with 50 nM OTS167 or gilteritinib. Phosphorylation levels of 4E-BP1 and eIF4B were assayed at 8 h by western blot (Fig. 3A – western blot) and quantitated by measuring band densities (Fig. 3A – graphs). Treatment of MV4:11 or MOLM-14 with OTS167 resulted in significant loss of both thr37/46 (**P < 0.01) and ser65 (***P < 0.001) phosphorylation of 4E-BP1. Whereas treatment with gilteritinib resulted in a partial non-significant loss of thr37/46 and a significant (**P < 0.01) loss of ser65 phosphorylation. Inhibitors of PIM kinase (AZD 1208) or mTORC1 (KU-0063794) were also tested for their ability to inhibit 4E-BP1 phosphorylation (Fig. S3). Neither was as effective as OTS167, nor able to downregulate FLT3-ITD, c-Myc or MCL-1 (Fig. S4). For eIF4B, treatment of MV4:11 or MOLM-14 with OTS167 resulted in a complete loss of ser406 and ser422 phosphorylation (****P < 0.0001), while treatment with gilteritinib resulted in a loss of ser422 phosphorylation (****P < 0.0001), but only a 50% decrease (**P < 0.01) of ser406 phosphorylation. To determine if inhibition of ser406 and ser422 phosphorylation occurs prior to FLT3-ITD downregulation, MV4:11 and MOLM-14 were treated with 50 nM OTS167 and assayed for eIF4B phosphorylation over time (Fig. S5A). Inhibition of ser422 phosphorylation occurred more rapidly than inhibition of ser406 phosphorylation. Treatment with 50 nM OTS167, gilteritinib or midostaurin all resulted in similar inhibition of ser422 phosphorylation, suggesting inhibition of a common pathway. After OTS167 treatment, phosphorylation of ser406 decreased at a similar rate to the loss of FLT3 protein expression (Fig. S5B). To determine if eIF4B activity is required for FLT3-ITD protein expression, MV4:11 or MOLM-14 cells were electroporated with eIF4B-siRNA and FLT3-ITD protein expression was assayed at 72 h by western blot (Fig. 3B – western blot). Collectively, the loss of eIF4B expression resulted in a significant (*P < 0.05) loss of FLT3-ITD expression, although the effect was more dramatic in MOLM-14 (*FLT3-ITD*^*+/-*^) than MV4:11 (*FLT3-ITD*^*+/+*^*)* (Fig. 3B – graph). OTS167 has demonstrated off-target effects resulting in inhibition of other cellular kinases^35^.(Fig. S6) To determine whether downregulation of FLT3-ITD expression was specifically due to inhibition of MELK activity, MELK knockdown using MELK-siRNA was performed in MV4:11 or MOLM-14 cells via electroporation and cells assayed at 48 and 72 h for their expression of FLT3-ITD and MELK protein (Fig. 3C – western blot). Knockdown of MELK protein expression resulted in a significant (**P < 0.01) decrease in FLT3-ITD expression in MV4:11 or MOLM-14 cells at 48 h, though the decrease was more pronounced in MOLM-14 (Fig. 3C – graph). MELK knockdown in MOLM-14 also resulted in downregulation of c-Myc, but not MCL-1 (Fig. S7). MOLM-14 cells are heterozygous for *FLT3-ITD* compared to homozygous in MV4:11 cells, potentially explaining the increased sensitivity of MOLM-14 to inhibition of the MELK-eIF4B pathway^36^. Alternately, translation of aberrantly expressed proteins (FLT3-ITD and c-Myc) in MOLM-14 cells may be more sensitive to disruption of MELK activity than in MV4:11.

### OTS167 inhibits FLT3-ITD kinase activity and downstream signaling in *FLT3* mutant AML cell lines

**Fig. 4:**
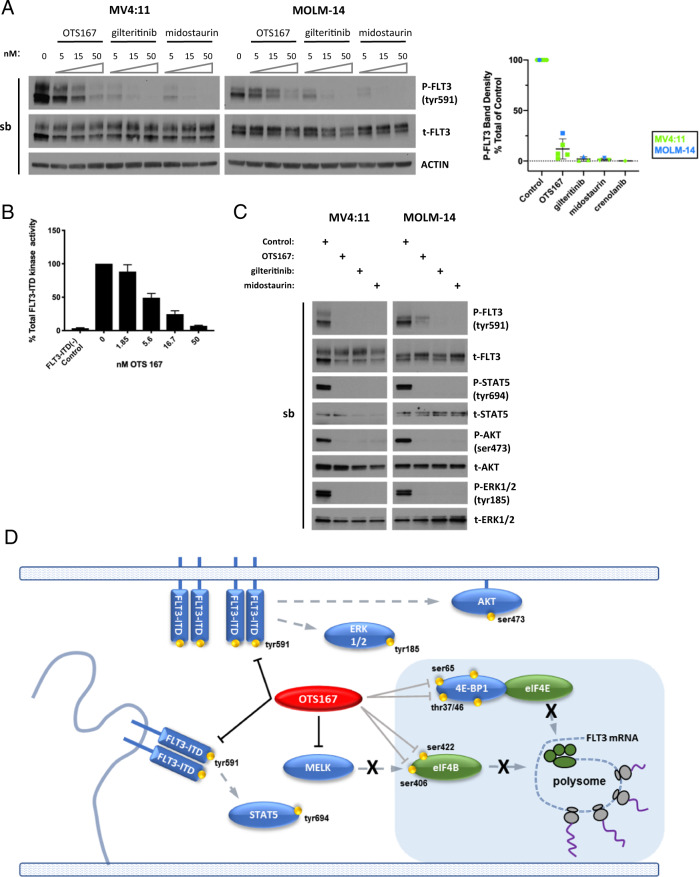
OTS167 inhibits FLT3 phosphorylation and signaling in *FLT3* mutant cell lines. **A** Western Blot analysis of P-FLT3 in MV4:11 or MOLM-14 after treatment with indicated concentrations of OTS167, gilteritinib, or midostaurin for 2 h. (7 biological replicates)(single blot). **B** In vitro kinase assay of FLT3-ITD recombinant protein with increasing concentrations of OTS167. (1 biological replicate; 3 technical replicates). **C** Western Blot analysis of P-STAT5, P-AKT, or P-ERK1/2 in MV4:11 or MOLM-14 after treatment with 50 nM OTS167, 50 nM gilteritinib, or 50 nM midostaurin for 2 h. (6 biological replicates)(single blot stripped and re-probed for total protein detection). **D** Schematic of signaling pathways inhibited by OTS167. Black blocking lines represent direct kinase inhibition. Gray blocking lines represent inhibition of downstream phosphorylation events. Dashed arrows represent signaling pathways.

**Fig. 5:**
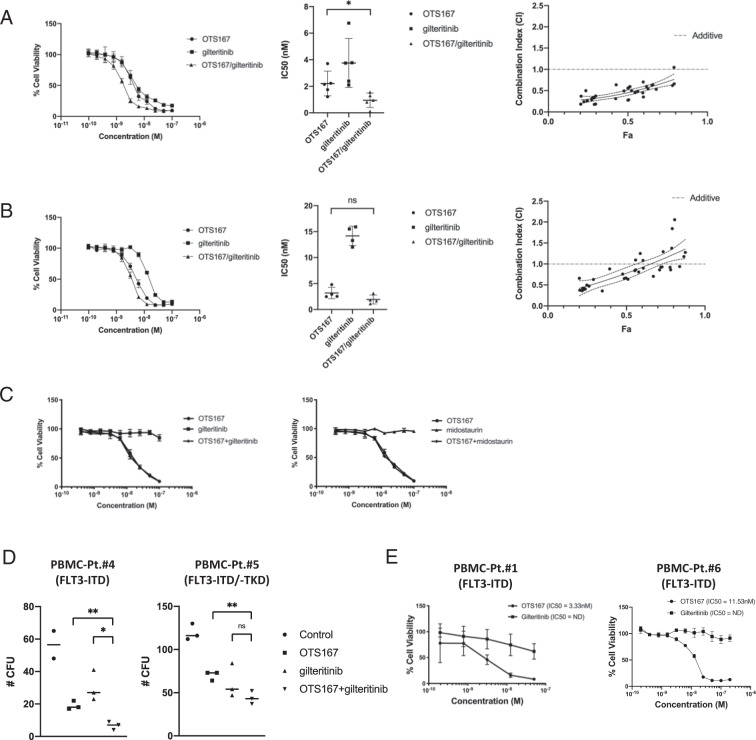
OTS167 acts synergistically with TKIs to induce cell death in *FLT3* mutant cell lines MV4:11 and MOLM-14. Representative cell viability assays (left panels), IC50 comparison (middle panels), and combination index (CI) graphs (right panels) for **A** MV4:11 and **B** MOLM-14 comparing single agent and combination (1:1) treatments at indicated doses. Combination index values were calculated using CompuSyn software. Combination index graphs (right panels) plot CI values at different cell viability percentages (Fa). Fa values between 0.2 (20% cell viability) and 0.8 (80% cell viability), representing the linear part of cell viability curves, are plotted. CI values <1 represent synergistic data points. Shown are linear regression curves with 95% confidence interval (dashed). IC50 comparison graphs represent 5 biological replicates (3 technical replicates) for MV4:11 (**A**) and 4 biological replicates (3 technical replicates) for MOLM-14 (**B**). Fa-CI plots (right panels) are individual data points (MV4:11 (*n* = 36); MOLM-14 (*n* = 37)) from 4 biological replicates for each cell line. **C** Representative cell viability assays for THP-1 (FLT3-WT) comparing indicated single agent and combination treatments. (2 biological replicates; 3 technical replicates). **D** Colony counts for colony-forming cell (CFC) assay using primary blasts obtained from two patients with *FLT3* mutant AML. Left graph sample treated with 4 nM OTS167, 4 nM gilteritinib, or 4 nM dose of both drugs. Right graph sample treated with 1 nM OTS167, 1 nM gilteritinib or 1 nM dose of both drugs. (1 biological replicate per patient; 2–3 technical replicates per dose). **E** Cell viability assays for primary blasts obtained from two patients with TKI-resistant *FLT3* mutant AML. (1 biological replicate per patient; 3 technical replicates per dose) (**P < 0.01, *P < 0.05, ns-not significant).

The constitutive phosphorylation of FLT3-ITD at tyr591 is known to activate downstream signaling mediated through the STAT5, MAPK, and PI3K pathways^37,38^. Inhibition of phosphorylation of FLT3-ITD by FLT3 kinase or PIM kinase inhibitors results in an increase of the 160 kDa complex glycosylated form of FLT3-ITD due to maturation of the 130 kDa form in the Golgi Apparatus, and greater sensitivity to FLT3 inhibitor-induced cell death^23,24,39^. An increase in the 160 kDa form of FLT3 observed 8 h after OTS167 treatment in *FLT3* mutant cell lines (Fig. 1A) suggested OTS167 might inhibit FLT3 tyr591 phosphorylation similarly. To determine whether OTS167 inhibits the constitutive phosphorylation of FLT3-ITD tyr591, MV4:11 or MOLM-14 were treated with increasing doses (5–50 nM) of OTS167, gilteritinib or midostaurin for 2 h. An OTS167 dose (50 nM) which induces maximal cell death at 48 h^17^ (Fig. 5A, B) inhibited phosphorylation of FLT3-ITD at tyr591 (Fig. 4A). OTS167 was also capable of directly inhibiting recombinant FLT3-ITD kinase activity with an IC50 of 4.9 nM (Fig. 4B). To assess whether OTS167 subsequently inhibits the signal transduction mediated by FLT3-ITD kinase activity, MV4:11 and MOLM-14 were treated with 50 nM OTS167, gilteritinib, or midostaurin and assayed by western blot for phosphorylation of STAT5, AKT, and ERK1/2 (Fig. 4C). OTS167 efficiently inhibited phosphorylation of the downstream signal transducers in both *FLT3* mutant cell lines similar to inhibition by FLT3-directed TKIs, further suggesting that OTS167 effectively inhibits FLT3 kinase activity. To determine the extent of OTS167 off-target inhibitory effects, a database of kinase inhibitors screened against common cellular kinases was queried. Results show that OTS167 inhibits several kinases involved in common leukemia cell signaling pathways (Fig. S6). The inhibition of FLT3-ITD-mediated signaling pathways supports a model whereby OTS167 inhibits both MELK- and FLT3-ITD-dependent signaling pathways necessary for efficient FLT3-ITD protein translation and kinase activity (Fig. 4D).

### OTS167 synergizes with FLT3 inhibitors in *FLT3* mutant AML and induces cell death in TKI-resistant primary blasts

Because OTS167 reduced FLT3 phosphorylation and protein levels in *FLT3* mutated cells, we asked whether this small molecule inhibitor might act synergistically with FLT3 inhibitors. MV4:11 or MOLM-14 were treated with increasing doses of OTS167, giltertinib, or a combination of equal doses of OTS167 and gilteritinib, and cell viability was measure after 48 h. Dose response curves demonstrate greater cell death with a combination of OTS167 and gilteritinib compared to each drug alone in MV4:11 or MOLM-14 (Fig. 5A, B, left panels). The average IC50 for MV4:11 was 0.95 nM for the combination OTS167 and gilteritinib compared to 2.20 nM and 3.76 nM for OTS167 or gilteritinib alone, respectively (*P < 0.5). For MOLM-14 an IC50 of 1.93 nM for the combination of OTS167 and gilteritinib was lower, but not statistically significant from 3.18 nM and 14.18 nM for OTS167 or gilteritinib alone, respectively. In MV4:11 cells treated with submaximal doses (5 nM) of drug, staining with Annexin V demonstrated that the cell death observed was due to apoptosis. An increased percentage of Annexin V positive events was seen after combination treatment with 5 nM OTS167 and 5 nM gilteritinib (36.0%) versus 5 nM OTS167 (21.1%) or 5 nM gilteritinib (20.8%) alone (Fig. S8). To quantitate the synergy between OTS167 and gilteritinib, the combination index (CI) was calculated using cell viability values from combination treatment and individual drug treatments^19^. CI values were plotted versus the experimental cell viability (Fa) induced. Fa values between 0.2 (20% cell viability) and 0.8 (80% cell viability) represent the linear (non-plateau) portion of the dose response curves and demonstrate a range of combination doses. CI values <1 represent a synergistic drug combination. In MV4:11 cells the CI values below 1 through the Fa range indicate a consistent synergism between OTS167 and gilteritinib to induce cell death (Fig. 5A right panel). For MOLM-14, CI values below 1 were observed at combinations of higher doses (lower cell viability) (Fig. 5B right panel). The cell line THP-1 expressing FLT3-WT was refractory to FLT3 inhibitors gilteritinib and midostaurin, suggesting the synergistic effect is limited to cells expressing mutant *FLT3* (Fig. 5C). Interestingly, the synergy between OTS167 and gilteritinib in MV4:11 cells was not augmented by ABT-199 (venetoclax), suggesting that priming of apoptosis in the MV4:11 cell line may not be Bcl-2-dependent (Fig. S9). Next, we examined the ability of OTS167 to induce cell death in *FLT3* mutant primary cells. To investigate the ability of OTS167 to inhibit *FLT3* mutant stem cell function, primary cells from two newly diagnosed *FLT3* mutant AML patients were tested in colony-forming cell assays in the presence of 1 or 4 nM OTS167, 1 or 4 nM gilteritinib or a combination of equal doses of both drugs. Single agent OTS167 was observed to inhibit AML blast colony formation at low nanomolar doses, synergizing with gilteritinib to inhibit colony formation in one of the two samples (Fig. 5D). Finally, in leukemia cells from two R/R *FLT3* mutant patients clinically resistant to TKIs (Table 1), whose cells were resistant to gilteritinib in vitro, treatment with OTS167 was capable of inducing cell death (Fig. 5E).

### Combination therapy of OTS167 with gilteritinib prolongs overall survival in a FLT3-ITD mouse model of *FLT3* mutant AML

**Fig. 6:**
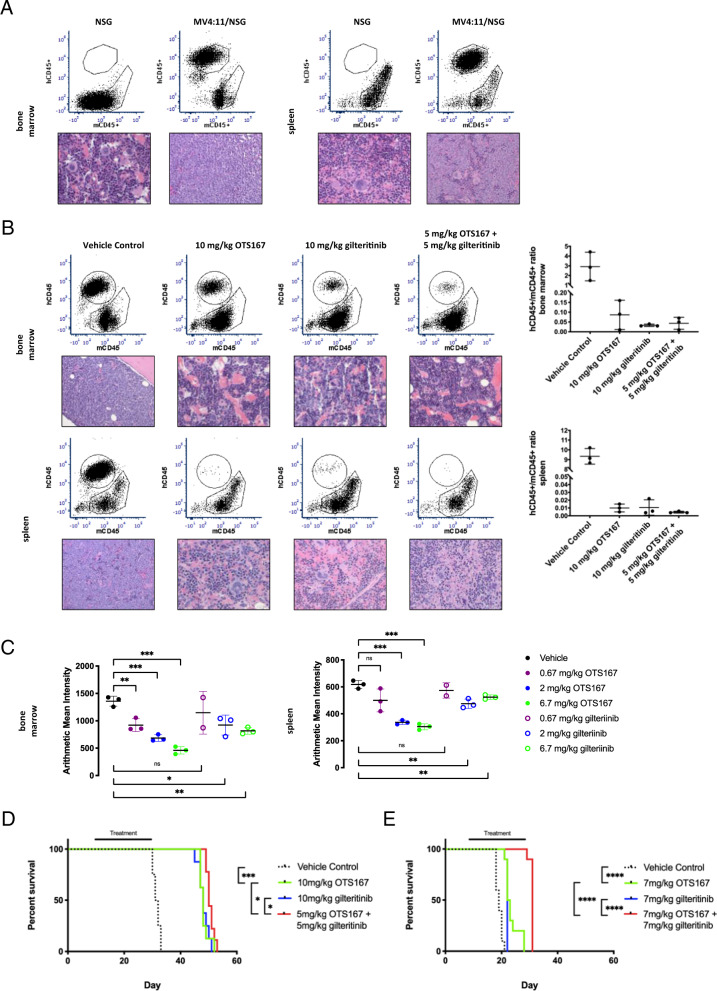
OTS167 acts synergistically to promote survival in a xenograft *FLT3* mutant AML mouse model. **A** Flow cytometry and histological analysis of bone marrow and spleen from representative NSG mice engrafted with MV4:11 (hCD45 + ). (3 biological replicates for flow analysis, 1 biological replicate for histology). **B** Flow cytometry and histological analysis of bone marrow and spleen from representative NSG mice engrafted with MV4:11 (hCD45 + ) after 14 days of indicated treatment. (3 biological replicates for flow analysis, 1 biological replicate for histology) Graphs show the ratio of hCD45 + /mCD45+ cells in bone marrow and spleen of NSG mice engrafted with MV4:11 based on flow cytometry analysis (from Fig. 6B). (3 biological replicates). **C** Flow cytometry analysis of bone marrow and spleen from NSG mice engrafted with MV4:11 (hCD33 + ) and treated (starting d10 via oral gavage) with vehicle (0.5% methylcellulose), OTS167 or gilteritinib at indicated doses. Graphs show arithmetic mean intensity of surface FLT3 staining (3 biological replicates (mice) per treatment group). **D** Survival analysis of MV4:11-engrafted NSG mice (N = 8–9 per group) treated (starting d10 via oral gavage) with vehicle (0.5% methylcellulose), OTS167, gilteritinib or OTS167, and gilteritinib at indicated doses. (8–9 biological replicates (mice) per treatment group). **E** Survival analysis of MOLM-14-engrafted NSG mice (*N* = 10 per group) treated (starting d7 via oral gavage) with vehicle (0.5% methylcellulose), OTS167, gilteritinib or OTS167 and gilteritinib at indicated doses. (10 biological replicates (mice) per treatment group). Histology images are ×500 (×10 ocular, ×50 objective). (****P < 0.0001, ***P < 0.001, *P < 0.05).

A previously described xenograft mouse model was used to test whether OTS167 acts synergistically with FLT3 inhibitors in an in vivo model of *FLT3* mutant AML^21^. Human *FLT3* mutant cell lines MV4:11 or MOLM-14 were injected via tail vein into NOD.Cg-*Prkdc*^*scid*^*IL2rg*^*tm1Wjl*^/SzJ (NSG) mice then treated with different doses of OTS167, gilteritinib, or a combination of OTS167 and gilteritinib based on the cell line tested. Flow cytometry and histological analysis of mice injected with MV4:11 cells (hCD45 + ) demonstrate engraftment of the MV4:11 cells in both bone marrow and spleen in untreated control mice (Fig. 6A). Histology of the tissues showed disease consistent with AML in the bone marrow and myeloid sarcoma in the spleen (Table S1-Vehicle Control). Initial studies determined the single-agent dose effectiveness of OTS167 and gilteritinib for mice engrafted with the MV4:11 or MOLM-14 cell lines (Fig. S10), which exhibit different degrees of aggressive disease. For treatment of MV4:11-engrafted mice (less aggressive disease; median survival 31.5 days), a high 10 mg/kg single-agent dose of OTS167 or gilteritinib was chosen and compared to a combination treatment of 5 mg/kg OTS167 and 5 mg/kg gilteritinib to represent an additive treatment. This MV4:11 dosing model could demonstrate a reduction of leukemia burden by OTS167 alone and in a combination with gilteritinib that represents an additive dose of each drug. A decrease of engrafted hCD45+ cells (MV4:11) was observed in both the bone marrow and spleen after single-agent treatment with 10 mg/kg OTS167, 10 mg/kg gilteritinib or a combination of 5 mg/kg OTS167 and 5 mg/kg gilteritinib and confirmed by histology (Fig. 6B and Table S1). Quantitation of flow cytometry images in Fig. 6B shows the ratio of hCD45 + /mCD45+ cells in the bone marrow and spleen (Fig. 6B – graphs). Surface FLT3 expression levels on MV4:11 cells from single-agent dose effectiveness studies using OTS167 or gilteritinib were also assessed by flow cytometry. MV4:11 cells isolated from the bone marrow and spleen after OTS167 treatment had reduced levels of FLT3 expression, demonstrating a dose-dependent in vivo downregulation of FLT3 by OTS167 (Fig. 6C). Under the same MV4:11 engraftment and dosing conditions we also tested the ability of the combination of the OTS167 and gilteritinib to improve overall survival of engrafted mice. Mice engrafted with MV4:11 and treated with a combination of 5 mg/kg OTS167 and 5 mg/kg gilteritinib (median survival 50 days) had improvement in overall survival that was statistically significant (*P < 0.05) compared to mice treated either with single-agent 10 mg/kg OTS167 (median survival 48 days) or 10 mg/kg gilteritinib (median survival 48 days)(Fig. 6D). The increased survival using a combined dose of 5 mg/kg each of OTS167 and gilteritinib compared with 10 mg/kg of each alone suggests that the resulting survival is not simply additive. Using the MOLM-14 cell line (more aggressive; median survival 19 days), a combination treatment of 7 mg/kg OTS167 and 7 mg/kg gilteritinib was compared to 7 mg/kg single-agent treatments. The MOLM-14 dosing model demonstrates a reduction of leukemia burden by OTS167 alone, and in combination with gilteritinib reproduces the synergistic results observed in cell viability assays in vitro (Fig. 5B). Mice engrafted with MOLM-14 and treated with a combination of OTS167 and gilteritinib (median survival 31 days) also had improvement in overall survival that was statistically significant (****P < 0.0001) compared with mice treated with single-agent OTS167 (median survival 22.5 days) or gilteritinib (median survival 22 days)(Fig. 6E).

## Discussion

In this study, we examined the antileukemic activity of the small molecule MELK inhibitor OTS167 in *FLT3* mutant AML. OTS167 is currently being evaluated in an early phase trial for patients with advanced myeloid malignancies. Efficacy of OTS167 against *FLT3* mutant cell lines suggested that OTS167 might act on mutant FLT3 through a MELK-dependent mechanism. Here we report two previously unrecognized effects of OTS167: (1) inhibition of FLT3 translation and (2) inhibition of FLT3-ITD kinase activity and downstream signaling. Recently OTS167 has been shown to inhibit the MELK-dependent phosphorylation of eIF4B in human breast cancer cell lines, which resulted in downregulation of anti-apoptotic factor myeloid cell leukemia 1 (MCL-1) expression^30^. In human multiple myeloma (MM), diffuse large B cell lymphoma (DLBCL) and mantle cell lymphoma (MCL) cell lines, MCL-1 expression was also inhibited by OTS167, although by an unknown mechanism^40,41^. Here we show OTS167 inhibits phosphorylation of eIF4B in human *FLT3* mutant cell lines, and in these cell lines loss of eIF4B expression results in the loss of FLT3-ITD expression. Cooperativity between eIF4B, eIF4A, and DEAD-box protein Ded1 for efficient translation of longer mRNA species supports the requirement of eIF4B for efficient expression of *FLT3*^31^. eIF4B has been shown to be phosphorylated on ser406 and ser422 via a combination of PI3K, MAP Kinase, Stat5, and MELK-regulated pathways, positively regulating activity^30,32,34,42^. By demonstrating the ability of OTS167 to inhibit multiple pathways mediated by FLT3-ITD activity, and the subsequent phosphorylation of eIF4B, we reveal the role eIF4B plays in FLT3-ITD protein translation. Our results show that eIF4B phosphorylation on both ser406 and ser422 was effectively inhibited by OTS167. These data support a model in which MELK contributes to phosphorylation of ser406 together with mTORC1, with additional mTORC1-mediated phosphorylation of ser422, culminating in activation of eIF4B^33^. In agreement with its ability to inhibit FLT3-ITD-mediated signaling pathways, OTS167 also inhibited phosphorylation of 4E-BP1. eIF4E, bound by hypo-phosphorylated 4E-BP1, is released and active after phosphorylation of 4E-BP1 first at thr37/46, then ser70 and ser65^29^. Interestingly, OTS167 was more effective than gilteritinib, a potent FLT3 kinase inhibitor, at inhibiting phosphorylation of 4E-BP1 at the two sites examined despite similar inhibition of PI3K signaling by the two drugs.

Known potential mechanisms of TKI resistance include up-regulation of FLT3L or FLT3 expression, activation of parallel pro-survival pathways, modulation of anti-apoptotic pathways, and additional *FLT3* point mutations^43^. Different juxtamembrane (JM) or activation loop mutations in the *FLT3* gene result in varying levels of constitutive FLT3 kinase activity, thus driving diverse levels of persistent signaling by the STAT5, PI3K, and MAPK pathways^38,44,45^. To deter resistance mutations, second-generation TKIs effective against both JM and activation loop mutations such as crenolanib, quizartinib, and gilteritinib are currently being tested in clinical trials, and gilteritinib has recently received FDA approval for treatment of relapsed *FLT3* mutant AML^11,46,47^. OTS167 inhibited the kinase activity of recombinant FLT3-ITD in vitro, and in AML cell lines. While the ability of OTS167 to inhibit FLT3 kinase activity and signaling was shared with other TKIs, only OTS167 is capable of downregulating FLT3 expression, circumventing resistance mechanisms including increased FLT3 or FLT3L expression and signaling in AML, or increased WT FLT3 expression in other hematologic malignancies such as *MLL(KMT2A)*-rearranged acute leukemias^48^.

The synergy between OTS167 and TKIs in combination treatment experiments in vitro and in vivo suggests that OTS167 may induce cell death in a manner other than through cooperative FLT3 kinase inhibition. Drugs targeting translation initiation factors eIF4A, eIF4E, 4E-BP1, as well as translation elongation have been developed and show encouraging results in several cancer models and clinical trials^49^. OTS167 treatment resulted in the downregulation of mutant FLT3 expression due to inhibition of FLT3 translation. Although not investigated here, additional effects of MELK inhibition may contribute to the induction of leukemia cell apoptosis, as observed in non-FLT3 mutant cell lines^17^. Furthermore, additional off-target effects of inhibitor OTS167 on other cellular pathways cannot be ruled out. While the mechanism of synergy is not fully understood, the ability of OTS167 to inhibit FLT3 kinase activity and downregulate FLT3 expression make it an attractive potential therapeutic option for *FLT3* mutant AML. Results from our *FLT3* mutant xenograft model demonstrate a synergy between OTS167 and gilteritinib in vivo, suggesting potential therapeutic value for OTS167 and a path forward for future dual agent targeting in *FLT3* mutant AML. Currently, dose-escalation in the OTS167 clinical trial is proceeding to determine a maximum tolerated dose and biologically effective dose that could be tested in combination therapy using OTS167 with gilteritinib in *FLT3* mutant AML.

## Supplementary information

Supplemental Material
