# Supplementary material for: OTS167 blocks FLT3 translation and synergizes with FLT3 inhibitors in FLT3 mutant acute myeloid leukemia
Source: Blood Cancer J. 2021 Mar 3;11(3):48. doi: 10.1038/s41408-021-00433-3 (PMC7930094; doi:10.1038/s41408-021-00433-3)
Supplement: Supplementary file 1 — Supplemental Material [file 41408_2021_433_MOESM1_ESM.pdf]

Supplemental Material:

Title: OTS167 Blocks FLT3 Translation and Synergizes with FLT3 Inhibitors in *FLT3* Mutant Acute Myeloid Leukemia

Authors: Bartholomew J Eisfelder[1], Caner Saygin[1], Joseph Wynne[1], Margaret W Colton[1], Mariafausta Fischietti[2], Elspeth M Beauchamp[2], Jason X Cheng[3], Olatoyosi Odenike[1], Gail Roboz[4], Houda Alachkar[5]* and Wendy Stock[1]*

Affiliations: *[1]Section of Hematology/Oncology, Department of Medicine, University of Chicago, Chicago, IL [2]Department of Hematology/Oncology, Robert H Lurie Comprehensive Cancer Center, Northwestern University, Chicago, IL [3]Department of Pathology, University of Chicago, Chicago, IL [4]Weill Cornell Medicine, The New York Presbyterian Hospital, New York, NY [5]Department of Clinical Pharmacy, School of Pharmacy, University of Southern California, Los Angeles, CA*

*These authors contributed equally to this work.

Keywords: FLT3, FLT3-ITD, MELK, OTS167, AML

Running Title: OTS167 Blocks FLT3-ITD Translation in *FLT3* Mutant AML

Financial Support: B.J. Eisfelder was supported by the V Foundation (#T2014-008).

Corresponding Author:
Bartholomew J Eisfelder
900 E 57th Street
KCBD #8112
Chicago, IL 60637

Conflict of Interest Statement:
O. Odenike, G. Roboz and W. Stock report receiving clinical research funding from OncoTherapy Science, Inc. (OTS) for conducting a Phase I clinical trial with OTS167. No potential conflicts of interest were disclosed by the other authors.

Supplemental methods

Cell Lines, Patient Samples and Reagents

All cell lines were maintained in RPMI supplemented with 10% fetal bovine serum (FBS)(Gemini Bio-Products #100-106, West Sacramento, CA). MV4:11 (*FLT3-ITD*$^{+/+}$) (RRID: CVCL_0064) were validated (CellCheck 9 Plus) and mycoplasma tested by IDEXX BioAnalytics (Columbia, MO) before initiating studies. MOLM-14 (*FLT3-ITD*$^{+/-}$) (RRID: CVCL_7916) were obtained from DSMZ (Brunswick, Germany) before initiating studies. THP-1 (RRID: CVCL_0006) were kind gifts from the Janet Rowley Lab repository (unvalidated). Frozen aliquots of validated, mycoplasma tested cell lines were thawed every 4 months to maintain line consistency. Peripheral blood or bone marrow samples were collected from patients with AML after obtaining informed consent in accordance with the Declaration of Helsinki and approved by the institutional review board of the University of Chicago. Mononuclear cells were isolated from bone marrow aspirates or peripheral blood by Ficoll-Hypaque gradient sedimentation. MELK inhibitor OTS167 for *in vitro* studies and *in vivo* treatment was a kind gift from OncoTherapy Science, Inc. (Kawasaki City, Japan). The following kinase inhibitors were used for in vitro studies: gilteritinib (ChemieTek #CT-GILT, Indianapolis, IN) and midostaurin (Sigma-Aldrich, #M1323). Gilteritinib from Astellas Pharma (Tokyo, Japan) was used for *in vivo* treatment. Cycloheximide was purchased from Sigma-Aldrich (#C4859, St. Louis, MO). Bortezomib (S1013), 3-methyladenine(3-MA) (S2767), AZD 1208 (S7104), KU-0063794 (S1226) and ABT-199 (S8048) were purchased from Selleck Chemicals (Houston, TX).

Cell Lysis and Immunoblotting

Cell lysates prepared with CelLytic M (Sigma-Aldrich) with NaV (New England BioLabs) and protease inhibitor cocktail (Sigma-Aldrich, St. Louis, MO). Lysates were run on 4-20% gradient gels (Bio-Rad Laboratories, Hercules, CA) and transferred to PVDF membranes (Bio-Rad) using a Bio-Rad Turbo Blot apparatus. Membranes were blocked with 5% powdered milk (Oxoid Ltd, Basingstoke, UK) or 5% bovine serum abumin (BSA)(Fisher Scientific, Waltham, MA) in tris buffered saline with Tween-20 (TBST)(Teknova Inc, Hollister, CA) specific to the primary antibody. For some experiments, membranes were stripped at 50C in stripping buffer (62.5 M Tris pH7.6), 2% SDS, 0.7% beta-mercaptoethanol), then re-probed as above. Band quantitation performed using ImageJ (SCR_003070). Briefly, mean gray value was measured in bands from grayscale images of western blots. After inverting pixel values to reflect darker pixel values as increasing, the background mean gray value was subtracted from band intensity, followed by normalizing by housekeeping gene

(Tubulin or Actin). Finally, the band intensity for each condition was calculated as a percent total of control cells or zero time point. Immunoblotting reagents were purchased from Cell Signaling Technology (Danvers, MA), Santa Cruz Biotechnology (Dallas, TX), Fisher Scientific, and Sigma-Aldrich.

Table of Reagents used for Immunoblotting

REAGENT	SOURCE	CATALOG #	RRID #
P-FLT3 (Y589/591) (30D4)	Cell Signaling Technology	3464	AB_2107051
FLT3	Santa Cruz	SC-480	AB_2104968
FLT3 (8F2)	Cell Signaling Technology	3462	AB_2107052
P-STAT5 (Y694) (D47E7)	Cell Signaling Technology	4322	AB_10544692
STAT5 (ST5-8F7)	Thermo Fisher	33-5900	AB_2533129
P-AKT (S473) (D9E)	Cell Signaling Technology	4060	AB_2315049
AKT (C67E7)	Cell Signaling Technology	4691	AB_915783
P-p44/42 MAPK (ERK1/2) (T202/Y204) (D13.14.4E)	Cell Signaling Technology	4370	AB_2315112
p44/42 MAPK (ERK1/2) (137F5)	Cell Signaling Technology	4695	AB_390779
P-eIF4B (S406) (D1C10)	Cell Signaling Technology	8151	AB_11178943
P-eIF4B (S422)	Cell Signaling Technology	3591	AB_2097522
eIF4B	Cell Signaling Technology	3592	AB_2293388
P-4E-BP1 (T37/46) (236B4)	Cell Signaling Technology	2855	AB_560835
P-4E-BP1 (S65) (D9G1Q)	Cell Signaling Technology	13443	AB_2728761
4E-BP1 (T37/46)	Cell Signaling Technology	9452	AB_331692
MELK	OncoTherapy Science	custom	
MCL-1	Santa Cruz	Sc-819	AB_2144105
c-Myc	Cell Signaling Technology	13987	AB_2631168
beta-Actin (AC-15)	Sigma-Aldrich	A1978	AB_476692
alpha-Tubulin (DM1A)	Cell Signaling Technology	3873	AB_1904178
Anti-Mouse IgG - HRP	Cell Signaling Technology	7076	AB_330924
Anti-Rabbit IgG - HRP	Cell Signaling Technology	7074	AB_2099233

Proteomic Analysis

Quantitative proteomic analysis (TMT-10plex) was performed by MSBioworks LLC (Ann Arbor, MI). Briefly, cell lysis was performed with 600µL of urea lysis buffer (8M urea, 50mM Tris.HCl pH8, 150mM NaCl) using a QSonica sonic probe, incubated at room temperature for 1hr with mixing at 1000rpm in an Eppendorf Thermomixer, then clarified by centrifugation at 10,000g for 10min at

25degC. 50ug of each lysate was reduced with 14mM dithiothreitol followed by alkylation with 14mM iodoacetamide, then digested with 20ug sequencing grade trypsin (Promega) at 37degC overnight. The digest was terminated with formic acid, then centrifuged at 10,000g for 10min.

Each digested sample was processed by solid phase extraction using an Empore C18 (3M) plate under vacuum (5inHg) and lyophilized. For the 10-plex, samples were reconstituted with 200mM HEPES and labeled with TMT labels (ThermoFisher Product # UL292368) by incubating in a Thermomixer for 1.5hr at 25degC and 300rpm. The reactions were quenched with addition of 5% hydroxylamine for 15min at 25degC. Equal amounts of labeled peptide were combined and processed by solid phase extraction using an Empore C18 (3M) 10mg C18 cartridge. 250ug of the labeled pooled sample was separated into 96 discrete fractions using an Agilent 1100 HPLC system equipped with a diode array and fraction collector. The HPLC column was a 2.1 x 150mm Waters XBridge column and peptides were eluted with a basic (pH10) reverse phase buffer system. Fractions were pooled and each pooled sample was lyophilized and reconstituted in 0.1%TFA to produce 12 samples for LC-MS/MS analysis.

10% of each pooled sample fraction (the equivalent of ~2.5μg of digested sample) was analyzed by nano LC-MS/MS with a Waters M-class LC system interfaced to a ThermoFisher Fusion Lumos. Peptides were loaded on a trapping column and eluted over a 75μm analytical column at 350nL/min; both columns were packed with Luna C18 resin (Phenomenex). Each fraction was analyzed with a 2hr gradient (24hrs total LC-MS/MS time). The mass spectrometer was operated using a custom MS3 method. MS scans were acquired in the Orbitrap at 120,000 FWHM resolution, MS2 scans were acquired in the ion trap using CID at 35% NCE, product ions were isolated using synchronized precursor selection (SPS) and fragmented using HCD at 65% NCE. MS3 scans were acquired in the Orbitrap at 50,000 FWHM resolution from *m/z* 100-500. A 2s cycle time was employed for all steps.

Data were processed with MaxQuant version 1.6.5.0 (Max Planck Institute for Biochemistry) which incorporates the Andromeda search engine. The Andromeda settings were as follows: Enzyme, Trypsin/P; Database, SwissProt Human; Fixed modification, Carbamidomethyl (C); Variable modifications, Acetyl (protein N-term), Oxidation (M); Missed cleavages, 2; Reporter ion tolerance, 0.003 Da. The MaxQuant output was further processed using Microsoft Excel. The false discovery rate of proteins and peptides was set to 0.01. The dataset was normalized by subtracting the median, and a fold-change threshold of 1.6 was used to determine differentially expressed proteins. All datasets were subject to a student's t-test and P values of less than 0.05 were considered statistically significant. For functional enrichment analysis a Welch's t-test (5% permutated FDR) was performed on control vs. 8hr data. A Fisher exact test was performed on the annotations associated

with the proteins identified as increased/decreased between conditions. Gene Ontology (GO) (www.geneontology.org), Corum (mips.helmholtz-muenchen.de/corum/#) and Kyoto Encyclopedia of Genes and Genomes (KEGG)(www.genome.jp/kegg/) analysis was applied. Each experimental condition for quantitative proteomic analysis was performed in triplicate.

Polysomal Profiling and Fractionation

Cells were washed twice with Dulbecco's phosphate buffered saline (DPBS) and then lysed in lysis buffer (0.5% Triton X 100, 0.5 % sodium deoxycholate, 5 mM Tris pH 7.5, 2.5 mM MgCl2, 1.5 mM KCl 100µg/ml cycloheximide, 2 mM DTT, protease inhibitor and 1U/µl RNase inhibitor). Lysates were then centrifuged at 20000 x g for 20 minutes at 4C and supernatants were collected and snap frozen in liquid nitrogen. To isolate ribosomal fractions, lysates were layered on a sucrose gradient of 5 to 50%. Samples were centrifuged at 4C for 120 minutes at 35000 rpm in a Beckman SW41-Ti rotor. Absorbance was measured at 254 nm continuously in an ISCO density gradient fractionator with the following settings: pump speed, 1.5 ml/min; fraction size, 10 drops per fraction; chart speed, 150 cm per hour; sensitivity, 1; peak separator, off; noise filter, 0.5 seconds. Fluorinert^{TM} FC-40 (Sigma-Aldrich #F9755) was used to set the baseline in an UA-6 detector for all experiments. Quantitative measurement (area under the curve) of monosomal and polysomal peaks was performed using ImageJ.

Kinase Assay

Kinase activity of recombinant FLT3-ITD (Life Technologies #PV6190, Carlsbad, CA) in the presence of OTS167 was measured using ADP-Glo Kinase Assay (Promega #V6930, Madison, WI). Recombinant FLT3-ITD was incubated for 30min in kinase reaction buffer (40mM Tris pH7.5), 20mM MgCl2, 0.1mg/mL BSA, 2mM DTT, 2mM MnCl2, 100uM NaF) with poly E4Y as a substrate. Luminescence was read with a Bio Tek Synergy H4 plate reader using Gen5 software (SCR_017317).

Flow Cytometry

Flow Cytometry analysis was performed using an LSR II or LSR-Fortessa 4-15 (BD Biosciences, Franklin Lakes, NJ). Annexin V staining was performed using eBioscience Annexin V Apoptosis Detection Kit APC (Invitrogen #88-8007, Carlsbad, CA). Splenocytes and bone marrow were harvested and 70um filtered before blocking with anti-mFcR mAb 2.4G2 and then staining with

excess primary or control antibodies. Stained cells were washed and run on above flow analyzers in the presence of propidium iodide (PI) or 4′,6-diamidino-2-phenylindole (DAPI) for live/dead cell discrimination. Events displayed were first gated as PI^- or $DAPI^-$ then as singlets (using doublet discrimination). Absolute numbers of CD45+ leukemia cells were calculated using CountBright absolute counting beads (Invitrogen #C36950) added to samples before flow analysis. Data analysis was performed using FCS Express (De Novo Software, Glendale, CA)(SCR_016431).

Table of Reagents used for Flow Analysis

REAGENT	SOURCE	CATALOG #	RRID #
PE anti Mo CD45.1 (A20)	BioLegend	110707	AB_313496
PE Mo IgG2a Isotype Control (MOPC-173)	BioLegend	400212	AB_326460
APC Mo anti Hu CD45 (HI30)	BD Biosciences	555485	AB_398600
APC Mo IgG1 Isotype Control (MOPC-21)	BD Biosciences	555751	AB_398613
AlexaFluor 647 anti FLT3 (SF1.340)	Santa Cruz	sc-19635	AB_626899
AlexaFluor 647 Mo IgG1 Isotype Control	Santa Cruz	sc-24636	AB_737215
PE anti Hu CD33 (WM53)	BD Biosciences	555450	AB_395843

RNAi Targeting and qPCR

Control and MELK siRNA were purchased from Sigma-Aldrich.**(1)** eIF4B siRNAs were purchased from Life Technologies. A Bio-rad Gene Pulser was used for transfection of cell lines under the following parameters: (single) square wave 180V pulse for 12ms. Quantitation of mRNA was performed with mRNA-specific Taqman assays (Applied Biosystems, Foster City, CA) using a LightCycler 480 (Roche, Basel, Switzerland) or QuantStudio 3 (Thermo Fisher Scientific) on cDNA generated from RNA isolated using STAT-60 (Amsbio, Abingdon, UK). Specific mRNAs were normalized first to ACTIN or GAPDH (Taqman assay) then to control samples to generate percent-of-total values.

Table of Reagents used for RNAi and mRNA Detection

REAGENT	SOURCE	CATALOG #
MISSION siRNA Universal Negative Control #1	Sigma-Aldrich	SIC001
MELK-1 (UGCAGCUAGAUAGGAUGUC)	Sigma-Aldrich	custom
eIF4B-3 (GCACAGUAACGAUGACGAUtt)	Ambion (Life Technologies)	4392412-s4573
eIF4B-4 (GUAUCGAGAUCGUUAUGAUtt)	Ambion (Life Technologies)	4392412-s4574
FLT3 Taqman Gene Expression Assay	Applied Biosystems	4331182-Hs00174690

ACTIN Taqman Gene Expression Assay	Applied Biosystems	4331182-Hs99999903
GAPDH Taqman Gene Expression Assay	Applied Biosystems	4331182-Hs02758991

Histology

H&E staining was performed on formalin-fixed paraffin-embedded (FFPE) sections. Femur and tibia samples for bone marrow were decalcified prior to embedding. Embedding, sectioning and H&E staining of formalin-fixed tissues was performed by the Human Tissue Research Core at the University of Chicago. Slides were reviewed by a board-certified pathologist (author J.X.C.) at the University of Chicago.

1. Alachkar H, Mutonga MB, Metzeler KH, Fulton N, Malnassy G, Herold T*, et al.* Preclinical efficacy of maternal embryonic leucine-zipper kinase (MELK) inhibition in acute myeloid leukemia. Oncotarget **2014**;5(23):12371-82 doi 10.18632/oncotarget.2642.

Supplemental Figures

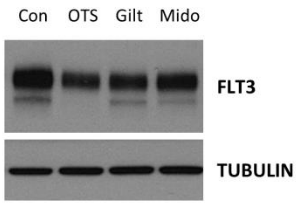

Figure S1. OTS167 downregulates FLT3-WT expression in MELK-expressing human AML cell line THP-1. Western blot analysis of FLT3 expression in THP-1 after treatment with 50 nM OTS167, 50 nM gilteritinib or 50 nM midostaurin for 24 hours. (single blot)

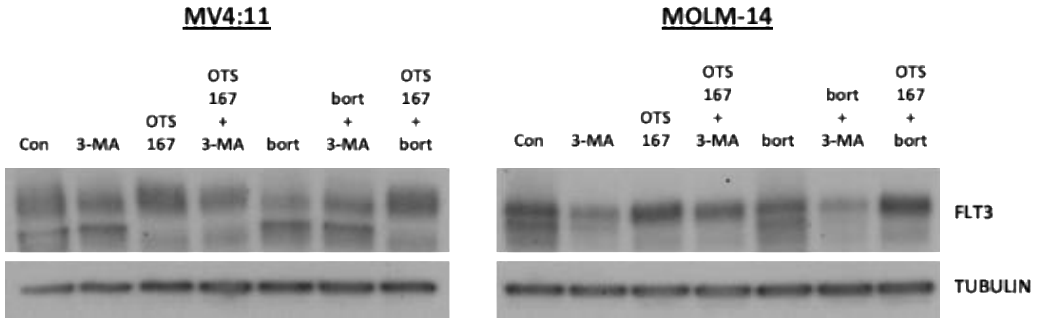

Figure S2. Downregulation of FLT3 by OTS167 is not inhibited by proteosome inhibitor bortezomib co-treatment in FLT3mut AML cell lines. Western blot analysis of FLT3 expression in MV4:11 and MOLM-14 after 18hrs treatment with 50nM OTS167, 5uM 3-MA, or 10nM bortezomib. (single blot)

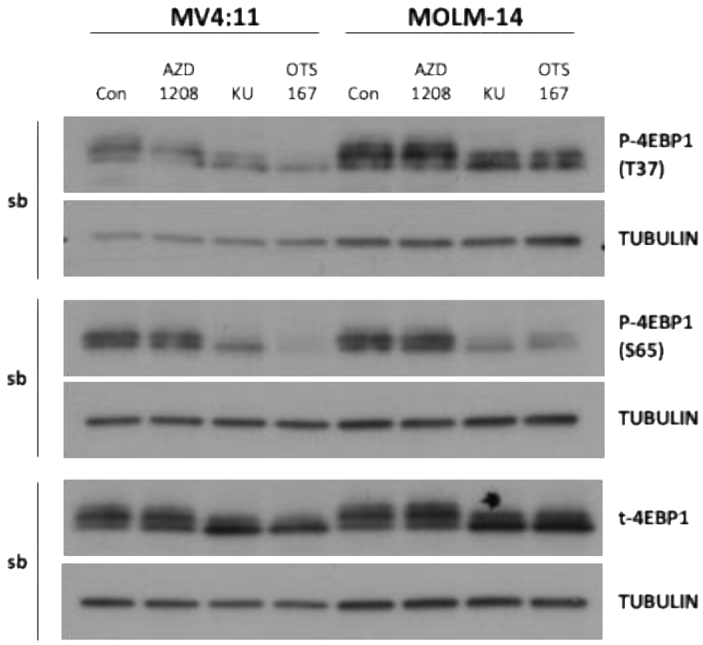

Figure S3. Inhibition of 4E-BP1 phosphorylation by pan-PIM kinase inhibitor AZD 1208 or mTORC1 inhibitor KU-0063794 in FLT3mut AML cell lines. Western Blot analysis of P-4E-BP1(thr37/46), P-4E-BP1(ser65) or t-4E-BP1 (4E-BP1 species detected in parallel blots) in MV4:11 or MOLM-14 after treatment with 1 uM AZD 1208, 1 uM KU-0063794 or 50 nM OTS167 for 8 hours.

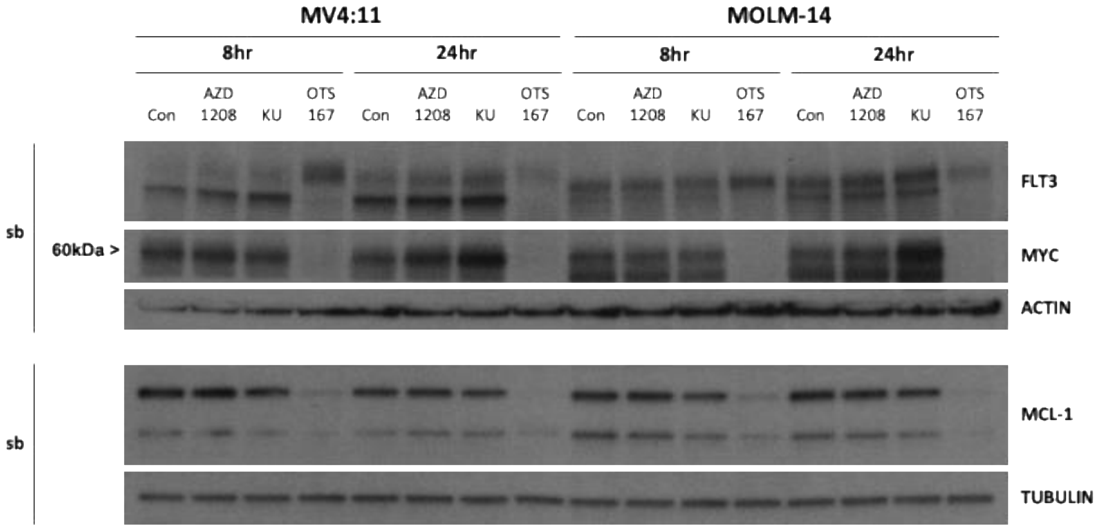

Figure S4. Effect of pan-PIM kinase inhibitor AZD 1208 or mTORC1 inhibitor KU-0063794 on expression of oncoproteins c-Myc and MCL-1 in FLT3mut AML cell lines. Western blot analysis

of FLT3, c-Myc and MCL-1 expression (FLT3 and c-Myc detected in separate blot from MCL-1) after treatment with 1 uM AZD 1208, 1 uM KU-0063794 or 50 nM OTS167 for 8 or 24hrs.

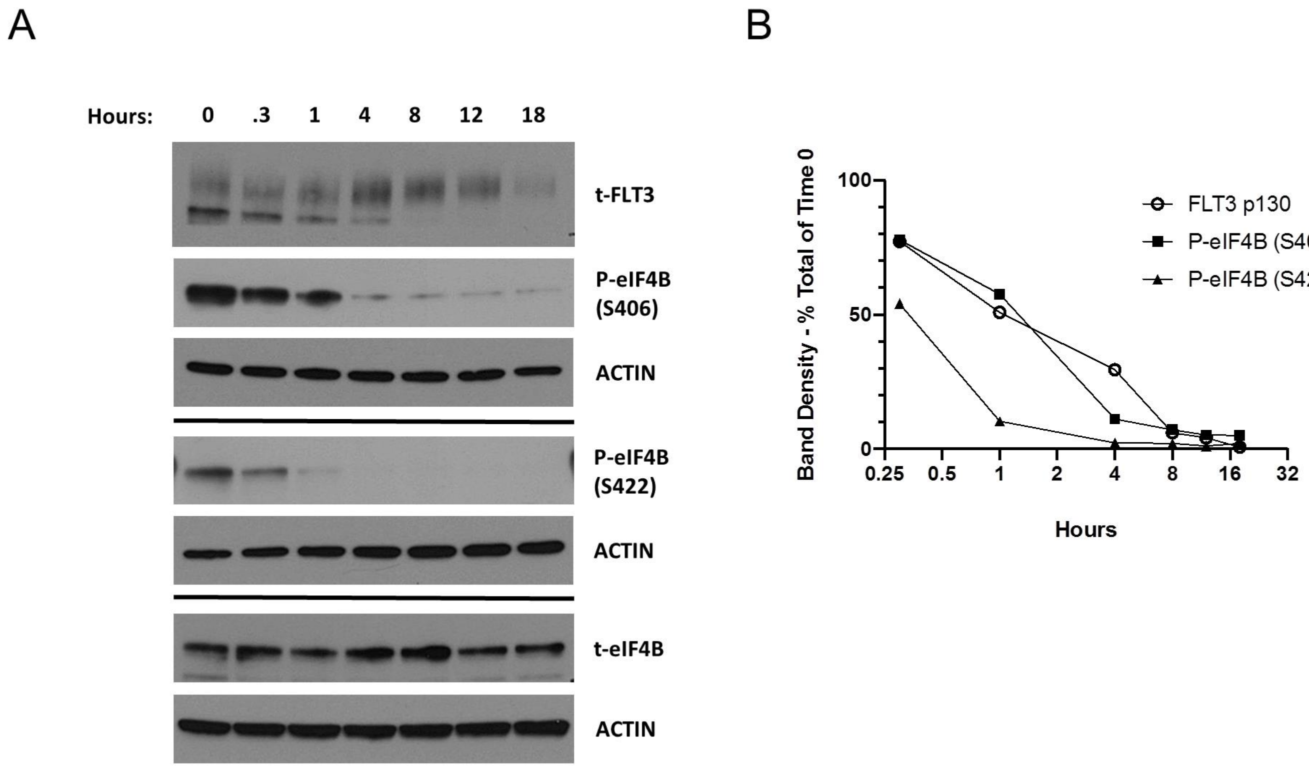

Figure S5. Time course of eIF4B phosphorylation in *FLT3*mut AML cell line MV4:11 after OTS167 treatment. A) Western Blot analysis of t-FLT3, P-eIF4B(S406), P-eIF4B(S422) or t-eIF4B (t-eIF4B and different phospho-eIF4B species were detected in parallel blots) in MV4:11 after treatment with 50 nM OTS167 for the indicated times. B) Graph of band densities (described in supplemental materials and methods) from Figure S2A. Band densities for each time point are normalized first to band density of Actin control, then to 0hr time point. (1 biological replicate)

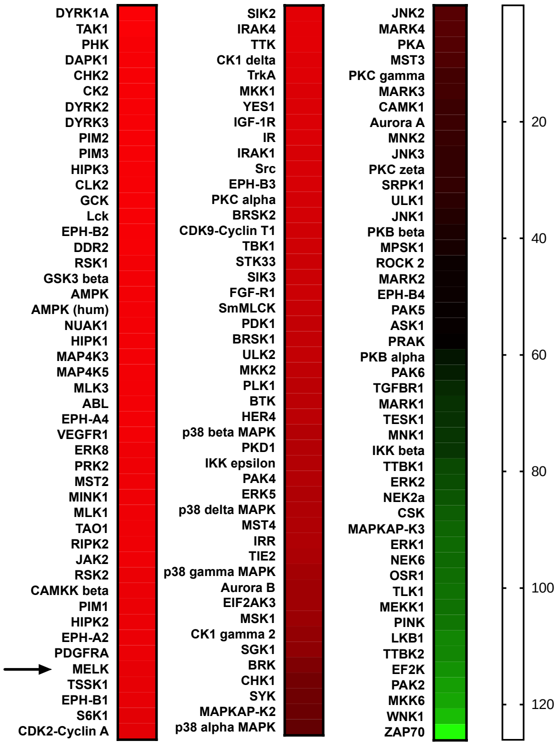

Figure S6. Heat Map of OTS167-inhibited kinases. Publicly available data from the Medical Research Council – Protein Phosphorylation Unit (MRC-PPU) at the University of Dundee (kinase-screen.mrc.ac.uk/kinase-inhibitors). Percentage activity remaining (heat map legend) measures *in vitro* kinase activity for the indicated kinase after incubation with 100nM OTS167. The lower the % activity remaining, the greater the specific kinase inhibition by OTS167. Black arrow highlights % activity remaining for MELK.

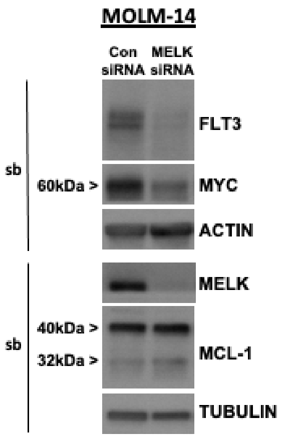

Figure S7. Effect of MELK knockdown on expression of c-Myc and MCL-1 in FLT3mut AML cell line MOLM-14. Western Blot analysis of FLT3, c-Myc, MELK and MCL-1 expression in MOLM-14 72hr after transfection with MELK-siRNA compared to Control-siRNA. (FLT3 and c-Myc detected in separate blot from MELK and MCL-1.)

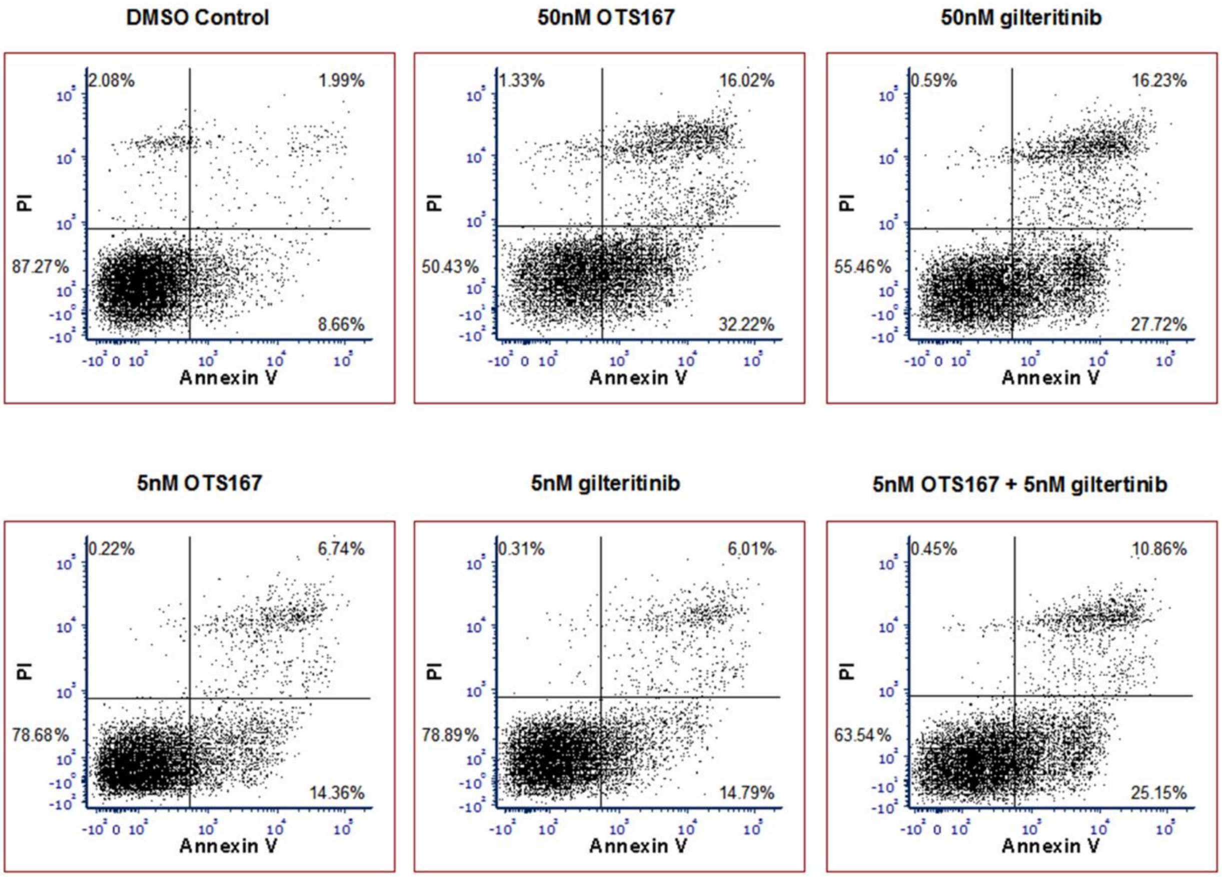

Figure S8. Increased induction of apoptosis by a combination of OTS167 and gilteritinib in *FLT3*mut AML cell line MV4:11. Flow cytometry analysis of MV4:11 cells treated with indicated concentrations of OTS167, gilteritinib or a combination of OTS167 and gilteritinib for 24hrs, then stained with Annexin V and propidium idodide (PI). (1 biological replicate)

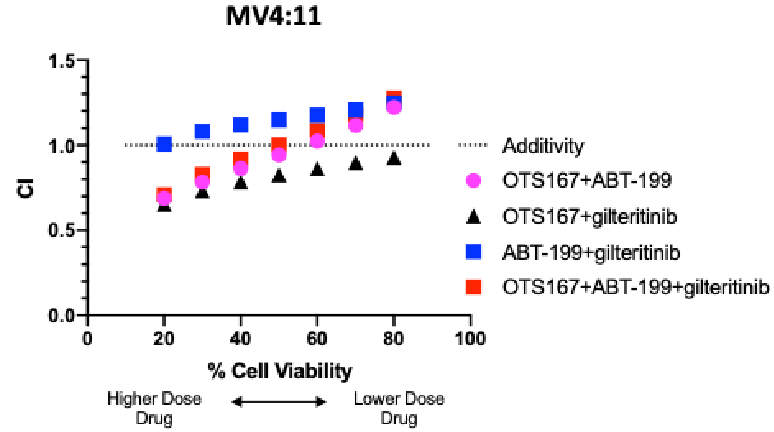

Figure S9. Combination treatments to induce cell death in *FLT3* mutant cell lines MV4:11 and MOLM-14. Combination index (CI) graph for MV4:11 comparing combination (1:1 or 1:1:1) treatments. Combination index graph plots CI values at different cell viability percentages (Fa). Fa values between 0.2 (20% cell viability and 0.8 (80% cell viability), representing the linear part of cell viability curves, are plotted. CI values <1 represent synergistic data points.

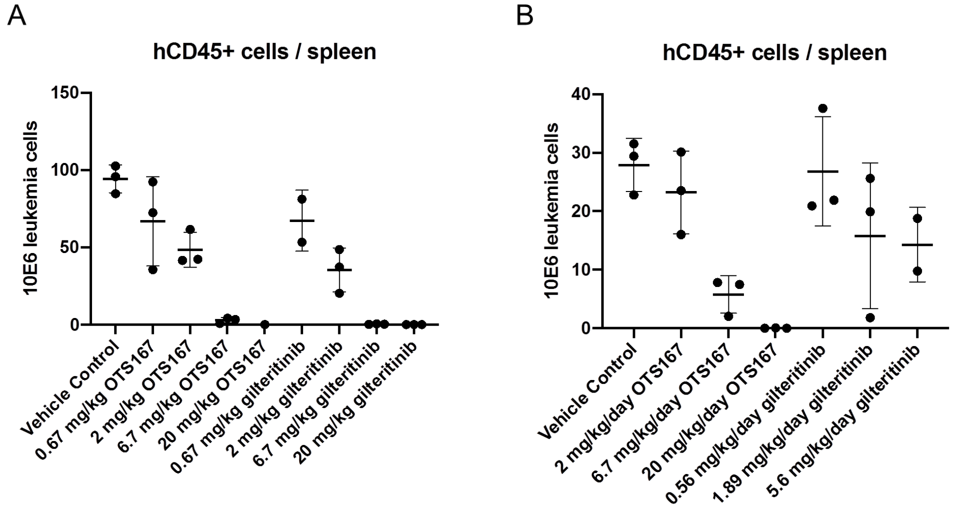

Figure S10. Determination of treatment doses for *FLT3*mut AML xenograft mouse model. Graph of the number of hCD45+ cells / spleen analyzed by flow cytometry in NSG mice engrafted with MV4:11 (A) or MOLM-14 (B) after 11 (MV4:11) or 5 (MOLM-14) days of indicated OTS167 or gilteritinib treatment. Treatment was started on day 10 after engraftment for both MV4:11 and MOLM-14 xenograft experiments. Absolute numbers of leukemia cells in each spleen were calculated using CountBright absolute counting beads added to samples before flow analysis. (1-3 biological replicates (mice) per dose)

Tissue	WT NSG	Vehicle Control	10 mg/kg OTS167	10 mg/kg gilteritinib	5 mg/kg OTS167 + 5 mg/kg gilteritinib
**bone marrow**					
Cellularity	95%	100%	65%	90%	85%
Blast	<5%	Markedly Incresed, 98-99%	Minimally Increased, 5-7%	~15%	Low level, 5-10%
Hemotopoiesis	Progressive	Abnormal	Progressive	ND	Progressive
Granulopoiesis	Predominant with many mature granulocytes	Rare mature granulocytes	Trilineage Hematopoiesis	Many mature granulocytes in focal areas	Trilineage Hematopoiesis
Erythropoiesis	Subset	Absent	Increased	Some	Increased
Megakaryopoiesis	Predominant	Absent	Predominant	Many megakaryocytes	Predominant
Diagnosis	Normal BM with trilineage hematopoiesis and granulocytic predominance	AML	Minimal Residual Disease	AML	Low level residual disease
**spleen**					
Blastic infiltrate	Not seen	95%	Minimal, 5%	No blastic infiltrate, <5%	Minimal, 5%
Extramedullary Hematopoiesis	Present, predominant	Minimal, <5%	Present, predominant	Present, predominant	Present, predominant
Lymphoid	Some lymphoid aggregates	Rare lympoid aggregates	Some lymphoid aggregates	ND	Some lymphoid aggregates
Diagnosis	No leukemic infiltrate, with extramedullary hematopoiesis	Myeloid Sarcoma	Minimal Involvement	No overt involvement	Minimal involvement

Table S1. Histology review of MV4:11-xenografted NSG mice treated with a combination of OTS167 and gilteritinib. Comments on hematoxylin and eosin (H&E) tissue slides from pathologist review of slides (slides reviewed from 1 mouse per experimental condition). Slides were generated from a representative MV4:11-engrafted NSG mouse after 14 days of the indicated treatment which started on day 10 after engraftment.
